# Fissure sealing and caries development in Norwegian children

**DOI:** 10.1007/s40368-022-00729-3

**Published:** 2022-07-07

**Authors:** H. B. Sæthre-Sundli, S. Y. Løken, N. J. Wang, T. I. Wigen

**Affiliations:** Department of Paediatric Dentistry and Behavioural Science, Institute of Clinical Dentistry, University of Oslo, Blindern, Box 1109, 0317 Oslo, Norway

**Keywords:** Children, Caries preventive treatment, Dental caries, Fissure sealants, Oral health

## Abstract

**Purpose:**

To explore the use of fissure sealing as a caries preventive method by describing characteristics of children who had received sealants and to study associations between sealing and caries prevalence at 12 years of age.

**Methods:**

The study included 3075 children examined at 5 and 12 years of age. Data were collected by clinical examination and questionnaire to parents. The questionnaire provided information on child characteristics and oral health behaviour in children at 5 years of age. Data on sealing and caries prevalence were obtained from dental records. Bi- and multivariate logistic regressions were performed. The Regional Committee for Medical Research Ethics approved the study (2.200.54 and 2013/1881).

**Results:**

Of the children, 12% had received sealants on permanent teeth before 12 years of age. Children with dentin caries at 5 years of age (OR 2.0, CI 1.5–2.7) had a higher probability of having received sealants than children without caries. Having sealants (OR 2.8, CI 2.2–3.6), enamel caries (OR 1.5, CI 1.2–1.9), dentin caries (OR 2.9, CI 2.3–3.6) and using fluoride lozenges less than daily (OR 1.5, CI 1.3–1.8) at 5 years of age were associated with having dentin caries prevalence at 12 years of age.

**Conclusion:**

Few children had received fissure sealing. Although sealing was used as a caries preventive method in children who had experienced caries in primary teeth, these children continued developing caries in their permanent teeth.

## Introduction

Keeping children’s teeth sound and caries free is a major goal in child dentistry (Pitts and Mayne 2021). Both home based and professional interventions may be used to prevent caries development. Homebased interventions such as tooth brushing with fluoridated toothpaste and limited sugar intake are recommended for all children, while fluoride supplements and professional interventions such as applying fluoride varnish and fissure sealing are mainly used in caries risk children (Banerjee et al. 2020; Chestnutt et al. 2017). These interventions have all been shown to be able to prevent caries development, however, it is still unknown if one intervention is superior to the other (Chestnutt et al. 2017; Hesse et al. 2021; Hilgert et al. 2015).

Dental caries in children is a global challenge (Wen et al. 2022). In the Scandinavian countries, caries prevalence in children has gradually decreased and the distribution in the dentition has changed with occlusal surfaces of molars being most frequently affected (Norderyd et al. 2015; David et al. 2006). Caries risk in molars is highest during the first years after the eruption, explained by morphology and posterior position (Mejare et al. 2004; Carvalho 2014). Fissure sealing has shown to prevent caries development in occlusal surfaces and be a cost-effective method both in caries prevention and in non-operative treatment of initial caries. In low-caries populations, sealing may be the preferred caries preventive method since most lesions are limited to pits and fissures (Ahovuo-Saloranta et al. 2017; Schwendicke et al. 2015).

Fissure sealing was introduced as a caries preventive method in the 1960s, with the intention to inhibit bacteria to accumulate in fissures (Wallis 1973). Sealing have been used to various degree, explained by differences both in caries prevalence and national guidelines on the use of sealants in addition to the organization of dental services provided to children (Leskinen et al. 2008). The reported proportion of children with sealants in Europe varies from 8 to 73% (Ekstrand et al. 2007; Leskinen et al. 2008; Panagidis and Schulte 2012; Pieper et al. 2013).

In Norway, dentine caries prevalence in children is low (Statistics Norway a 2022). Children are regularly examined in the dental services, and caries preventive treatment provided is based on individual assessment. Fissure sealing has been recommended as one of the preventive methods in caries risk children since 1999 (The Norwegian Directorate Of Health 1999). Limited information exists on the use of sealing as a preventive method in children and the association between sealing and caries prevalence.

The objective of the study was to explore the use of fissure sealing as a caries preventive method by describing the characteristics of children who had received sealants and to study associations between sealing and caries prevalence at 12 years of age.

## Materials and methods

All children in Akershus county in Norway (*n* = 7002) were invited to participate as part of routine dental examination at 5 years of age. In total, 5623 children (80%) were included and 3282 were available for re-examination at 12 years of age (Saethre-Sundli et al. 2020; Løken et al. 2019). In 207 children, data on sealing was not present. No statistically significant difference was found between children with or without data on sealing, regarding dentin caries (16% vs 14%), non-Western background (14% vs 10%) and parental low education (42% vs 42%). Children with incomplete data were excluded and the final population consisted of 3075 children.

### Clinical examination

Data were collected by clinical and radiographic examination in dental clinics as part of the regular dental examination in the dental services. Information about the sealing of permanent teeth was extracted from dental records, and children categorized as having received sealants or not. Caries were registered at 5 and 12 years of age, and lesions both in enamel and dentin were included. Radiographs were used as an adjunct to the clinical caries registration when visual inspection of approximal surfaces was impossible and used in 75% of the children at the 5-year examination and in 97% at the 12-year examination. At 5 years of age, children were categorized as having no caries, only enamel caries and dentin caries. At 12 years of age, children were categorized as having no caries and dentin caries. The clinical dental examination of the children was performed by 44 dental hygienists at the 5-year examination and 46 hygienists and 45 dentists at the 12-year examination.

Using Cohen’s kappa, intra- and interexaminer agreements were tested based on 20 bitewing radiographs of primary molars including 8 approximal surfaces in each radiograph before the examination at 5 years of age (Landis and Koch 1977). Mean intra- and interexaminer values were 0.85 (SD 0.12) and 0.86 (SD 0.10). Before examination at 12 years of age, intra- and interexaminer agreements were based on 8 radiographs of permanent molars including 12 approximal surfaces in each radiograph. The mean intra- and interexaminer values were 0.69 (SD 0.16) and 0.69 (SD 0.17).

### Questionnaire

Child characteristics and oral health behaviour were retrieved from a questionnaire completed by parents at the 5-year examination.

Child characteristics included information on gender, parental nationality and education. Parental background was recorded as country of birth, categorized as mother and father having Western or non-Western background. Variables were combined and dichotomized into both parents with Western background and one or both with non-Western background. Non-Western background included parents born in Asia, Africa, South America, Central America and Eastern Europe. Parental education was recorded as mother and father years of education. High education was defined as 12 years or more and low education was defined as less than 12 years at school. Variables were combined and dichotomized into both parents with high education and one or both parents with low education.

Child’s oral health behaviour at 5 years of age included information on tooth brushing, use of fluoride lozenges and frequency of sugar snacking. Tooth brushing was reported as twice daily, once daily and sometimes, dichotomized into brushing twice daily and less than twice daily. Use of fluoride lozenges was reported as daily, less than daily and never, dichotomized into daily and less than daily. Sugar snacking included sugary drinks, cakes, biscuits and candy, reported as less than once a week, once a week, several times a week and several times a day, dichotomized into once a week or less and several times a week or more.

### Statistical analyses

The statistical analyses were conducted using IBM SPSS Statistics for Windows, Version 27.0. Armonk, NY, IBM Corp. Spearman’s rank correlation was calculated to study collinearity between independent variables. Bivariate and multivariate logistic regression analyses were conducted using fissure sealing and dentin caries prevalence at 12 years of age as dependent variables. Data were presented by using frequency and odds ratio (OR) with 95% confidence interval (CI). The level of significance was set at 5%.

### Ethical approval

Written, informed consent was obtained from parents at both examinations. The Regional Committee for Medical Research Ethics approved the study (2.200.54 and 2013/1881).

## Results

Table 1 shows the characteristics of children. At 12 years of age, 12% of the children had received sealants on permanent teeth and 32% had dentin caries experience. At 5 years of age, 11% of the children had enamel caries and 15% had dentin caries experience. The majority of children had both parents with Western background, brushed their teeth twice daily and used fluoride lozenges daily.

**Table 1:** Characteristics of children (*n* = 3075)

	%	(*n*)
Fissure sealing		
No	88	(2720)
Yes	12	(355)
Caries at 12 years of age		
No caries	68	(2720)
Dentin caries	32	(997)
Gender		
Boy	52	(1586)
Girl	48	(1489)
Parental background		
Both Western	86	(2661)
One or both non-Western	14	(414)
Parental education		
Both high	58	(1781)
One or both low	42	(1294)
Tooth brushing^a^		
Twice daily	74	(2271)
Less than twice daily	26	(800)
Fluoride lozenges^a^		
Daily	69	(2120)
Less than daily	31	(942)
Sugar snacking^a^		
Once a week or less	46	(1397)
Several times a week or more	54	(1607)
Caries at 5 years of age		
No caries	74	(2259)
Only enamel caries	11	(334)
Dentin caries	15	(482)

^a^Reduced because of internal dropout

Table 2 presents results from the bi- and multivariate logistic regression analyses exploring associations between child characteristics, oral health behaviour, caries at 5 years of age and fissure sealing. An association was found between sealing and caries prevalence at 5 years of age and gender. The multivariate analysis showed that children more likely to have received sealing were those with dentin caries at 5 years of age (OR 2.0, CI 1.5–2.7) and girls (OR 1.3, CI 1.0–1.6).

**Table 2:** Bi- and multivariate logistic regression analyses exploring associations between child characteristics, oral health behaviour, caries at 5 years of age and children with sealants

	Children with sealants			
	Bivariate (*n* = 3075)		Multivariate (*n* = 2989^a^)	
	OR	(95% CI)	OR	(95%CI)
Gender				
Boy (ref) Girl	**1.2**	**(1.0–1.5)**	**1.3**	**(1.0–1.6)**
Parental background				
Both Western (ref) One or both non-Western	1.0	(0.7–1.4)	0.8	(0.6–1.1)
Parental education				
Both high (ref) One or both low	**1.3**	**(1.0–1.6)**	1.2	(1.0–1.5)
Tooth brushing^a^				
Twice daily (ref) Less than twice daily	1.2	(0.9–1.5)	1.2	(0.9–1.5)
Fluoride lozenges^a^				
Daily (ref) Less than daily	0.8	(0.6–1.0)	0.8	(0.6–1.0)
Sugar snacking^a^				
Once a week or less (ref)				
Several times a week or more	1.0	(0.8–1.3)	1.0	(0.8–1.3)
Caries at 5 years of age No caries (ref)				
Only enamel caries Dentin caries	1.1 **2.0**	(0.7–1.5) **(1.5–2.6)**	1.1 **2.0**	(0.7–1.6) **(1.5–2.7)**

Statistical significant differences marked in bold

*Ref* reference

^a^Reduced because of internal drop-out

Table 3 shows results from bi- and multivariate logistic regression analyses exploring associations between fissure sealing, child characteristics, oral health behaviour, caries at 5 years of age and dentin caries prevalence at 12 years of age. The multivariate analysis showed that children who had received sealants had 2.8 times higher probability of having dentin caries at 12 years of age than children without sealants. Children more likely to have dentin caries at 12 years of age, were those with enamel caries (OR 1.5, CI 1.2–1.9), dentine caries (OR 2.9, CI 2.3–3.6) and who used fluoride lozenges less than daily (OR 1.5, CI 1.3–1.8) at 5 years of age.

**Table 3:** Bi- and multivariate logistic regression analyses exploring associations between children with sealants, child characteristics, oral health behaviour, caries at 5 years of age and dentin caries prevalence at 12 years of age

	Children with dentin caries			
	Bivariate (*n* = 3075)		Multivariate (*n* = 2989^a^)	
	OR	(95% CI)	OR	(95% CI)
Fissure sealing				
No (ref)				
Yes	**3.0**	**(2.4–3.7)**	**2.8**	**(2.2–3.6)**
Gender				
Boy (ref)				
Girl	1.0	(0.9–1.2)	1.0	(0.9–1.2)
Parental background				
Both Western (ref)				
One or both non-Western	**1.7**	**(1.4–2.1)**	1.1	(0.9–1.4)
Parental education				
Both high (ref)				
One or both low	**1.4**	**(1.2–1.6)**	1.1	(1.0–1.3)
Tooth brushing^a^				
Twice daily (ref)				
Less than twice daily	**1.3**	**(1.1–1.5)**	1.1	(1.0–1.4)
Fluoride lozenges^a^				
Daily (ref)				
Less than daily	**1.6**	**(1.4–2.0)**	**1.5**	**(1.3–1.8)**
Sugar snacking^a^				
Once a week or less (ref)				
Several times a week or more	**1.2**	**(1.0–1.4)**	1.0	(0.9–1.2)
Caries at 5 years of age				
No caries (ref)				
Only enamel caries Dentin caries	**1.6** **3.5**	**(1.2–2.0)** **(3.0–4.3)**	**1.5** **2.9**	**(1.2–1.9)** **(2.3–3.6)**

Statistical significant differences marked in bold

*Ref* reference

^a^Reduced because of internal drop-out

## Discussion

The study aimed to describe the use of fissure sealing as caries preventive method in children and to explore associations between sealing and caries prevalence. The results showed that sealing of permanent teeth was used in children having caries in primary teeth. Children having received sealants more often developed caries in permanent teeth.

Proportion of children with sealants was low, at the same time in line with some European studies (Leskinen et al. 2008; Panagidis and Schulte 2012). The low use of fissure sealing may be explained by caries prevalence, as a proportion of children with sealants was similar to children who had dentin caries in primary teeth. Another explanation may be that dental personnel considered sealing as a difficult treatment in newly erupted molars and relied on other caries preventive methods, such as fluoride varnish and oral hygiene instructions (Uhlen et al. 2019; Michalaki et al. 2010). In Scandinavia, the dental treatment is free of charge and dental personnel are salaried. Choosing sealing as a treatment has no financial incentive in comparison to some other European countries (Michalaki et al. 2010; Leskinen et al. 2008).

The result from this study suggests that dental personnel considered children with past dentin caries experience as caries risk children and chose to apply sealants to prevent caries in permanent teeth. Past caries experience is one of the predictors often used when assessing caries risk before deciding to apply sealants (Splieth et al. 2010; Welbury et al. 2004). Enamel caries in primary teeth and oral health behaviour were not associated with the use of sealants in this study. This may suggest that dental personnel did not rely on enamel caries and oral health behaviour in caries risk assessment before choosing to seal as a caries preventive method.

An association between fissure sealing and caries prevalence at 12 years of age was found. Children who had received sealing were caries risk children and continued developing caries despite the use of sealants. The results from this study support previous studies showing that sealing alone does not reduce caries development and should be applied in addition to other caries preventive methods (Splieth et al. 2010; Ekstrand et al. 2007; Hilgert et al. 2015; Chestnutt et al. 2017).

In this study, an association between enamel caries in primary teeth and caries prevalence at 12 years of age was registered. Enamel caries in primary teeth can be used as a predictor for further caries development in permanent teeth (Isaksson et al. 2013). The results suggest that enamel caries in primary teeth could be included in caries risk assessment when choosing caries preventive method in children.

The present study was a follow-up of a large group of children over a period of 7 years. In longitudinal studies, non-participation and dropout may cause selection bias. Caries prevalence in the studied population was on the same level as the national average (Statistics Norway b 2022), and results were assumed to be representative of the country in general.

Some of the limitations of the study would be recall bias, misconceptions and answering in a social desirable way due to the questionnaire used in the study. However, the questions were considered to be uncomplicated relating to oral health behaviour and child characteristics, thus minimizing report errors (Sjöström et al. 1999). This study was not designed to evaluate sealing material or technique nor to evaluate fissure sealing at the tooth level. The clinical examinations were performed by experienced dental personnel and the calibration showed substantial to almost perfect agreement (Landis and Koch 1977).

This study showed that fissure sealing was used in children with the highest caries risk. The results suggest that enamel caries and oral health behaviour were not included in caries risk assessment before sealants were applied. Increased focus on caries risk assessment and caries prevention in children seem to be warranted to prevent caries development in newly erupted permanent teeth. The collection of data took place prior to the establishment of a new national guideline for dental services to children that have enhanced focus on caries prevention and the use of fissure sealing (The Norwegian Directorate of Health 2018). Future studies will show if the present recommendation augments the use of sealants and results in a further decrease in caries prevalence.

## Conclusion

Considering the limitations of the present study the following conclusions can be made:

- Few children had received fissure sealing.
- Sealing was used as caries preventive method in children who had experienced dentine caries in primary teeth.
- Children having received sealants continued developing caries in permanent teeth.
- Sealing alone does not reduce caries development and should be combined with other caries preventive methods.
